# Effects of diabetes mellitus on amyotrophic lateral sclerosis: a systematic review

**DOI:** 10.1186/1756-0500-7-171

**Published:** 2014-03-24

**Authors:** Alain Lekoubou, Tandi E Matsha, Eugene Sobngwi, Andre P Kengne

**Affiliations:** 1 Neurological and Neurosurgical Hospital, Lyon, France; 2 Department of Biomedical Technology, Faculty of Health and Wellness Sciences, Cape Peninsula University of Technology, Cape Town, South Africa; 3 National Obesity Centre, Yaounde Central Hospital and Faculty of Medicine and Biomedical Sciences, University of Yaoundé 1, Yaoundé, Cameroon; 4 South African Medical Research Council & University of Cape Town, Cape Town, South Africa; 5 The George Institute for Global Health, Sydney, Australia; 6 Julius Center for Health Sciences and Primary Care, University Medical Center Utrecht, Utrecht, The Netherlands

## Abstract

**Background:**

Amyotrophic lateral sclerosis (ALS) is an incurable motor neuron degenerative disease which onset and course may be affected by concurrent diabetes mellitus (DM). We performed a systematic review to assess the effect of DM/dysglycemic states on ALS.

**Methods:**

We searched PubMed MEDLINE, from inception to March 2013 for original articles published in English and in French languages on DM (and related states) and ALS. We made no restriction per study designs.

**Results:**

Seven studies/1410 citations (5 case–control and 2 cross-sectional) were included in the final selection. The number of participants with ALS ranged from 18 to 2371. The outcome of interest was ALS and DM/dysglycemic states respectively in three and two case control-studies. DM/impaired glucose tolerance status did not affect disease progression, survival, disease severity and disease duration in ALS participants but ALS participants with DM were found to be older in one study. DM/IGT prevalence was similar in both ALS and non ALS participants. This review was limited by the absence of prospective cohort studies and the heterogeneity in ALS and DM diagnosis criteria.

**Conclusions:**

This systematic review suggests that evidences for the association of ALS and DM are rather limited and derived from cross-sectional studies. Prospective studies supplemented by ALS registries and animal studies are needed to better understand the relationship between both conditions.

## Background

Diabetes mellitus (DM) is a common condition affecting 347 million people worldwide [1]. It is associated with complications such as renal failure, coronary heart disease, stroke, limb amputation and peripheral neuropathies. Neuropathies are the most common complications of DM, affecting up to 50% of diabetic patients [2,3]. The deleterious effect of diabetes on peripheral nerves results from the interplay of defects in metabolic and vascular pathways alongside oxidative stress [4]. Furthermore, diabetes mellitus may increase peripheral nerve susceptibility to a wide range of physical and metabolic agents or even accelerate other disease processes affecting peripheral nerves. *Put together, these epidemiological and mechanistic evidences suggest that DM may play a role in both initiation and progression of peripheral nerve injuries in a wide variety of pathological conditions*. Those conditions include diseases with worse prognosis such as amyotrophic lateral sclerosis (ALS). ALS remains an incurable disease and about fifty percent of those affected will die within 3 years from symptoms onset. Although ALS has been described since over a century, the exact mechanistic pathways leading to the progressive degeneration of motor neuron remain incompletely understood [5]. The current pathophysiologic conception is that of an interplay of genetic factors (multiple genes), developmental, environmental and age-related factors leading to pathological changes and ultimately loss of motor neurons [6]. Characterising the effects of diabetes mellitus on ALS occurrence has the potential of improving the understanding of ALS pathology and opening new therapeutic avenues.

We aim to conduct a systematic review of published evidence on the association between diabetes mellitus and amyotrophic lateral sclerosis.

## Methods

### Data sources and strategies

We searched PubMed MEDLINE, from inception to March 2013 for articles published in English and French languages on diabetes (and related states including glucose intolerance, hyperinsulinemia) and the risk of amyotrophic lateral sclerosis. We included the term “fronto-temporal dementia” (FTD) in the search strategy as there are compelling evidence to suggest that FTD and ALS are clinical expressions of the same disease spectrum [5].

Search strategy included the following terms: ("Diabetes" OR "Diabetes mellitus" OR “Pre-diabetes” OR “Prediabetes” OR “Glucose intolerance” OR “Hyperinsulinemia” OR “Hyper-insulinemia” OR “hyperglycemia”) AND (“neurodegeneration” OR “neurodegenerative diseases” OR “neurodegenerative disorders” OR “fronto-temporal dementia” OR “FTD” OR “amyotrophic lateral sclerosis” OR “ALS” OR “motor neuron disease” OR “Charcot disease” OR “Lou Gehrig disease” OR "frontotemporal lobe dementia") NOT ("animal"). In addition, we manually searched the reference lists of eligible articles and relevant reviews, and traced studies that had cited them through the ISI Web of science to identify additional published and unpublished data. Two evaluators (AL and APK) independently identified articles and sequentially screened them for possible inclusion. Eligible articles had to report on diabetes and its association/impact on ALS in human subjects. We made no restriction by study design, thus case-series, hospital-based and population-based studies were considered for inclusion. Disagreements were solved by consensus.

### Data extraction

Two investigators (AL and APK) independently conducted data extraction and quality assessment. From each study, we extracted data on study setting, study design, study population characteristics, diagnostic criteria for ALS and diabetes mellitus/pre-diabetes states, measure of disease (ALS and diabetes mellitus) occurrence (incidence/prevalence) and measure of association between ALS and diabetes mellitus (Odd ratio and Relative risk).

### Data synthesis

Given the wide range of measures of association between DM/pre-diabetes and ALS across studies, as well as the variety of study designs, we opted to conduct a narrative analysis without a formal meta-analysis.

## Results

**Figure 1:**
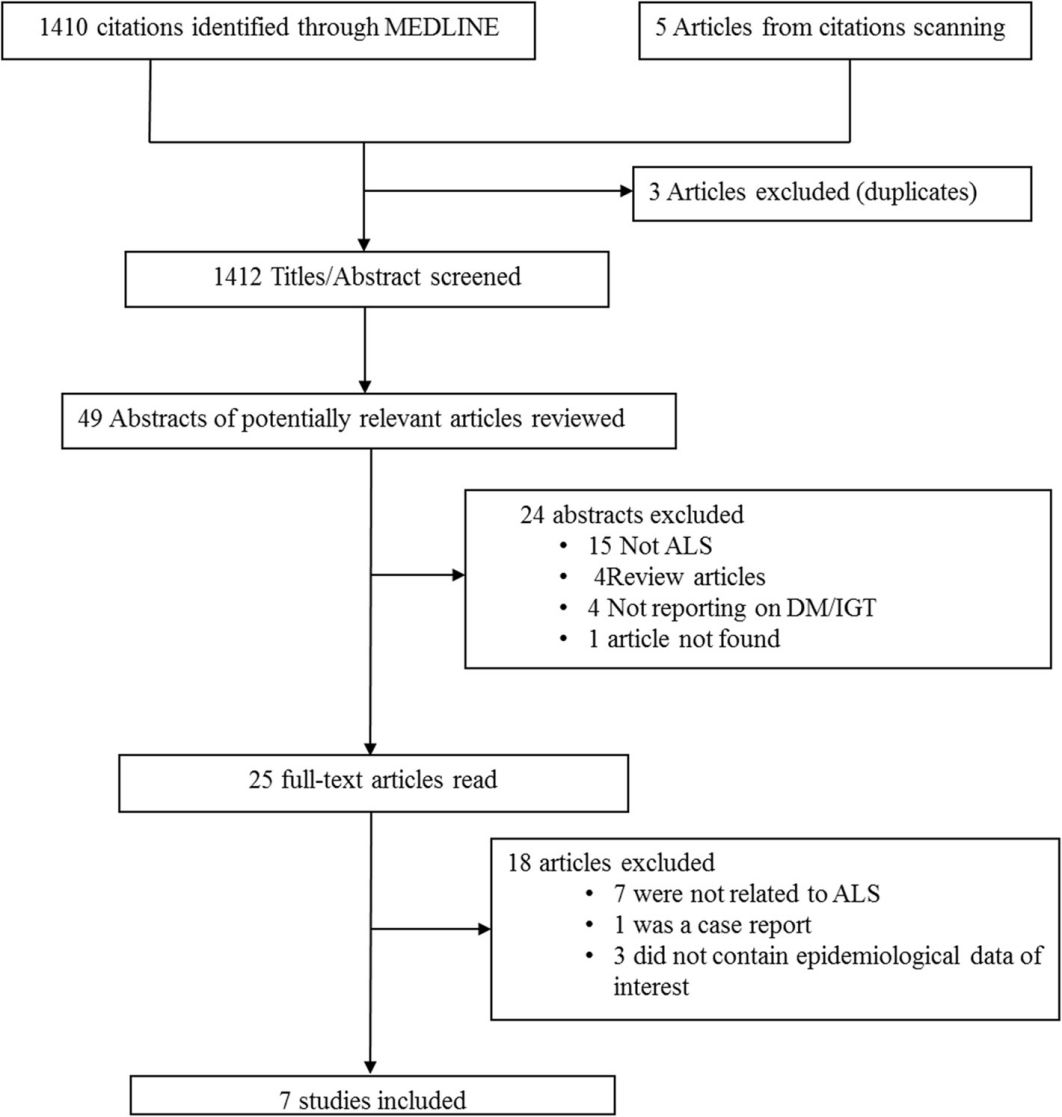
Flow chart for the study selection.

Figure 1 describes the study selection process. Of the 1410 articles retrieved, 49 abstracts were selected for in-depth evaluation (with most articles being excluded for not reporting on ALS but rather on other neurodegenerative diseases mainly dementia and Parkinson’s disease) and 25 full texts were reviewed, of which 7 were included in the final selection. Descriptive data and main results are summarized in Table 1. Five were case–control and two were cross-sectional studies. All the seven studies were conducted in western countries including 4 in the USA, 1 in France, Romania and Finland each. The overall study size varies from 18 to 2371 participants with ALS. Diagnosis of ALS was based on EL Escorial criteria in 3 studies [7-9], on clinical criteria only in one study [10], on the combination of clinical and electromyographic (EMG) criteria in one study [11]; and it was not reported in two studies [12,13]. Likewise the diagnosis of diabetes mellitus and impaired glucose tolerance varies across studies. Two studies used the WHO 1997 criteria [8,9], one study use the United states public health service and the national diabetes data group criteria [12], while diagnosis criteria for diabetes mellitus and dysglycemia were not provided in 3 studies.

**Table 1:** Features of studies included in the final review

**Authors, Year of publication**	**Country setting**	**Design period of study**	**Population characteristics**	**ALS diagnostic criteria**	**Diabetes mellitus and pre-diabetes states diagnostic criteria**	**Outcomes of interest**	**Comments including association between DM and risk of ALS onset, progression or survival**
Jawaid [7], 2010	USA Hospital-based	Case–control	N: 274	El Escorial criteria	Not provided	Rate of progression beta coefficient (95% CI): -0.07 (-2.40-0.74), P =0.30	Adjustment performed for possible confounders including BMI change, gender, APoE genotype and Site of onset,
		1999-2004	Age: 52y			Survival beta coefficient (95% CI) 0.10 (-0.93-3.49) P =0.25	
			M: 171				
			Patient with pre-morbid DM were compared with patients without pre-morbid DM				
Jawaid [8], 2010	USA Hospital-based	Retrospective Case–control 1984-2007	Cases (ALS with pre-morbid DM):	El Escorial criteria	Diabetes mellitus	Age of onset in years: ALS with DM 60.3 vs. ALS without DM: 56.3 (p < 0.02)	Adjustment performed for possible confounders including gender and site of onset
			N: 175		Two FBG ≥ 126 mg/dl or two RBG ≥ 200 mg/dl at or before the time of ALS diagnosis.	Rate of progression (AALS/month): ALS with DM 3.58 vs. ALS without DM: 3.01 (p: NS)	
			Age: 60y			Survival (years): ALS with DM: 3.60 vs. ALS without DM 3.04 (p: NS)	
			M: 62				
			Control (ALS without pre-morbid DM):				
			N: 2196				
			Age: 56y				
			M: 66				
Pradat [9], 2010	France Hospital-based	Case–control	ALS:	El Escorial criteria	75 g OGTT	Prevalence of IGT in ALS vs. control (33% vs. 9.5% p =0.13)	OGTT compared between ALS patients and controls.
			N: 21 (including 7 with IGT)		DM: FPG > 7.0 mmol/l or the 2-h post-load blood glucose concentration > 11.0 mmol/l.	Disease duration ALS with IGT vs. ALS without IGT (17 months vs. 20 months, p = 0.62),	No adjustment performed for possible confounders with IGT vs. ALS without IGT (17 months vs. 20 months, p = 0.62),
			Age: 53y		IFPG: FPG between 6.1 and 7.0 mmol/l.	ALSFRS ALS with IGT 35 months vs. ALS without IGT 35 months, p = 0.89)	
			M: 86%		IGT: FPG < 7.0 mmol/l and 2-h blood glucose of 7.8-11.0 mmol/l		
			Control (non ALS):				
			N: 21				
			Age: 53y				
			M: 86%				
Ionacescu [10], 1968	Romania Hospital based	Cross-sectional	N: 18 participans with ALS	Clinical diagnosis: signs of peripheral motor neuron disease in upper and lower limbs + pyramidal signs	Not provided	Abnormal OGTT: 50%	
			Age: 52y			Exaggerated sensitivity to insulin: 61%	
			M: 10				
Koerner [12], 1976	USA Hospital-based	Retrospective Cross sectional	N: 34 participants with ALS	NA	USPHS criteria/100 g glucose load:	56% of ALS patient had an IGT or DM	Authors reported that the frequency of IGT and DM in ALS participants was higher than in other Asian pacific regions and USA
					IGT: 2-hour post glucose load > mean + 2SD		
			Age: NA				
			M: NA				
Harno [11], 1984	Finland Hospital based	Case–control	Cases:	Clinical and ENMG signs of lower motor neuron disease. Signs of upper motor neuron disease could be present	Diabetes: FPG of ≥140 mg/dL (7.8 mmol/L) or a 2-hour PG ≥ 200 mg/dL in an OGTT	Diabetes:	No adjustment for possible confounders
			N: 21		IGT: FPG <7.8 mmol/l, PG-1 h >11.1 mmol/l, PG-2 h 7.8-11.0 mmol/l)	Case: 5%	
			Age: 59y		PG-1 h >11.1 mmol/l, PG-2 h 7.8-11.0 mmol/l)	Control: 10%	
			M: 14			OR = 0.45 (95% CI: 0.03-8.02)	
			Control			Abnormal OGTT:	
			N: 10			Case: 19%	
			Age: 61y			Control: 20%	
			M:2			OR = 0.94 (95% CI: 0.14-6.25)	
Armon [13], 1991	USA Population-based	Retrospective Case–control 1925-87	N: 45	NA	Not provided “Diabetes as diagnosed and treated by physicians”	Diabetes:	No adjustment for possible confounders
			Age: 68y			OR = 1 (0.29-3.5)	
			M:51%			Case: 13%	
			Controls			Control: 13%	
			N: 90				
			Age: NA				
			M: NA				

ALS: Amyotrophic lateral sclerosis, DM: Diabetes mellitus, N: sample size, M: male sex, 95% CI: 95% confidence interval, BMI: Body mass index, FBG: Fasting blood glucose, RBG: Random blood glucose, FPG: Fasting plasma glucose, OGTT: Oral glucose tolerance test, IGT: Impaired glucose tolerance test, IFPG: Impaired fasting plasma glucose, PG: Plasma glucose, ENMG: Electroneuromyography, ALSFRS: Amyotrophic lateral sclerosis functional rating scale, AALS: Appel amyotrophic lateral sclerosis score, RR: relative risk, NA: Not available, y: years.

### Case control studies

In three studies, the outcome of interest was related to ALS [7-9]; these studies reported on the association between diabetes mellitus/IGT status in ALS and the rate of progression (2 studies), survival rate (2 studies), age of onset (1 study), disease duration (1 study) and disease severity (1 study). In two other studies [11,13], the rate of diabetes mellitus/IGT was compared between ALS and non ALS participants. DM/IGT status did not affect disease progression, survival, disease severity and disease duration in ALS participants but ALS participants with DM were found to be older in one study. DM/IGT prevalence was similar in both ALS and non ALS participants.

### Cross-sectional studies

Two cross-sectional studies reported a prevalence of IGT/DM ranging from 50 to 56% among ALS participants [10,12]. In one study, the authors reported that IGT/DM prevalence was higher in ALS compared to the general population in the same region.

## Discussion

According to this systematic review, diabetes mellitus and dysglycemia appear to be more frequent among patients with ALS, but do not seem to affect progression of disease, disease severity, disease duration and survival in these patients. Evidences however are either very limited or mostly derived from single institution, retrospective, case control and cross-sectional studies. Diagnosis criteria for ALS and DM/IGT vary substantially across existing studies, reflecting different time periods where those studies were conducted. For instance the EL Escorial criteria for ALS diagnosis were adopted in the year 2000 while 4 of the 7 included studies were conducted before 2000. Similarly, DM/IGT diagnosis criteria have been revised a few times in recent decades.

The lack of an association between DM/IGT and ALS reported in this review seems to be inconsistent with the known deleterious effect of DM on peripheral nerves. Similarly, studies looking at the association between ALS status/survival and metabolic abnormalities closely related to DM such as obesity and hyperlipidaemia have yielded conflicting results while in animal models, hypermetabolism and mitochondrial dysfunction observed in ALS suggest a link between energy expenditure, adiposity and motor neuron degeneration [14-18]. ALS patients are usually thin at least at the advanced stage of the disease possibly, as the result of poor oral intake (due at least in part to dysphagia) and hypermetabolism, suggesting interplay between metabolic abnormalities and ALS onset or progression. A few reasons could explain this apparent paradox including methodological issues. Most studies were case control, retrospective, cross-sectional or single-centre and there were significant heterogeneity in definitions of exposure and outcome variables across studies. Furthermore sample sizes were likely not large enough to reliably capture any sizable association. ALS is a multifactorial diseases whereby genetic, developmental and environmental factors interplay, resulting in loss of both primary and secondary motor neurons [5]. While the association between DM and ALS has not been rigorously tested in humans, there are suggestions from animal models that DM/IGT may interfere with ALS onset or progression through several mechanisms such as reactive oxidative species-mediated glucotoxicity or by the mean of neuro-inflammation. For instance, superoxide dismutase 1 (SOD1) mutation has been described in several families with ALS. SOD1 dysfunction which results in an increased intra-cellular reactive oxygen species also plays a major role in diabetes neuropathy. Animal models of ALS also suggest a role of neuroinflammation in the events leading to motor neuron loss and disease progression including microglial dysregulation, an increased secretion of pro-inflammatory markers such as INF-γ, TNF-α, a decreased secretion of protective cytokines such as IL-4, Th1-lymphocytes infiltration and Th-2 depletion [5,19-21]. The concept that DM is an inflammatory states and that it precisely induces the same metabolic abnormalities support the hypothetic role of diabetes in the pathogenesis and progression of ALS [22-25]. However it is worth recalling that most animal models have been derived from the transgenic mouse model overexpressing SOD1 (which is non diabetic but rather lean and hypermetabolic) while only 2% of ALS are related to this mutation [5]. Interestingly, more candidates genes including TDP-43 gene, FUS gene and the most recent and most common C9orf72 gene expansion, which phenotype is distinctively different from other forms of ALS have been reported more frequently; but animal models-when they exist- do not mimic clinical phenotypes of ALS as closely as SOD1 mouse [5].

While investigating the relationship between DM and ALS has the potential of shedding light on some pathological and therapeutic aspects of ALS, these efforts may nevertheless be hindered by several shortcomings. Evidence from such an association would be best derived from prospective studies; however the short survival rates among ALS participants would make such a study particularly difficult to conduct. In addition, achieving large sample size comparable to those of common complex diseases like DM would be a challenge for a rare disease such as ALS. Collaborative efforts would hopefully overcome this specific challenge. Beside the difficulties related to conducting prospective studies, ALS has not received the same consideration as other neurological diseases which may explain why after more than a century there are very few intervention/drugs that alter the clinical course of the disease. Fortunately, the recent growing interest of the scientific community has led to the development of animal models-though the complexity of the disease would require more models- which may contribute to improve among others, the understanding of the relationship between DM and ALS. Likewise, ALS registries are important components of a multi-level strategy and would include data on diabetes mellitus status. Lastly, criteria for ALS diagnosis, ALS severity scoring and diabetes mellitus have changed across years, therefore making comparisons across studies challenging. There is a need to standardize current ALS diagnosis criteria which would include merging data from the most widely used criteria i.e. the El Escorial and the Airlie House criteria. Likewise, it is suggested that the amyotrophic lateral sclerosis functional rating scale (the most widely used score for assessing disease severity and progression in ALS) would not be linear in early and late stage of disease hence, this should be accounted for in statistical analysis [26]. New candidate variables will need to be evaluated. Disease-severity scales deserve to be revisited and standardized; for instance recent findings that those with dysexecutive functions progress more rapidly suggest that a clinical classification based on cognitive function at diagnosis and rate of progression would be more useful than the traditional upper/lower motor neuron and site of involvement classification [27].

To the best of our knowledge, this is the first attempt to systematically summarize data on the association between ALS and DM. In line with the recent trends, our systematic review may increase awareness among ALS scientists on the need to define subtypes of ALS and probe into common mechanistic pathways between diabetes mellitus and ALS. We are well aware of the limitations of our review. First, the wide heterogeneity between studies designs and outcomes precluded a refined summary of results and particularly the ability to perform a meta-analysis. Second, evidences were derived uniquely from cross-sectional studies and case–control studies therefore, making it impossible to establish the sequence of occurrence or an attempt to draw a mechanistic relationship between ALS and DM. Third, we could not assess publication biases.

## Conclusions

In conclusion, this systematic review suggests that evidence for the association of ALS and DM are rather limited and derived from observational studies. Prospective studies supplemented by ALS registries and animal studies are needed to better understand the relationship between both conditions. Those studies should ideally focus on common mechanistic pathways between ALS and DM such as increased intracellular reactive species, mitochondrial dysfunctions, inflammation as well as pathways through which DM may induce neurodegneration in other parts of the CNS. Such pathways include among other defects in insulin production, insulin resistance and clustering of cardiovascular risk factors and their neuro-toxic effects. Findings from these studies may ultimately pave the way to improving the dread prognosis of ALS.

## Competing interests

The authors declare that they have no competing interest.

## Authors’ contributions

AL took part in the study conception, did the literature search, study selection and data extraction, and drafted the manuscript. TEM took part in the study design and critically revised the manuscript. ES took part in the study design and critically revised the manuscript. APK took part in the study conception, study selection and data extraction and helped to draft the manuscript. All authors read and approved the final manuscript.
